# Crimean–Congo Hemorrhagic Fever Mimicking HELLP Syndrome in a Pregnant Woman and Her Infant in Kosovo: A Case Report

**DOI:** 10.3390/v17020178

**Published:** 2025-01-26

**Authors:** Lindita Ajazaj-Berisha, Bahrije Halili, Vera Ndrejaj, Kurtesh Sherifi, Xhevat Jakupi, Simone Priesnitz, Christoph J. Hemmer, Salih Ahmeti, Petra Emmerich

**Affiliations:** 1 Infectious Diseases Hospital, University Clinical Center of Kosovo, 10000 Prishtina, Kosovo; dr_lindita@yahoo.com (L.A.-B.); barie_halili@hotmail.com (B.H.); vera.ndrejaj@hotmail.com (V.N.); 2 Faculty of Agriculture and Veterinary, University of Prishtina “Hasan Prishtina”, 10000 Prishtina, Kosovo; kurtesh.sherifi@uni-pr.edu; 3 National Institute of Public Health of Kosovo, 10000 Pristina, Kosovo; xhevat.r.jakupi@rks-gov.net; 4 Bundeswehr-Krankenhaus (German Armed Forces Hospital), Lesserstraße 180, 22049 Hamburg, Germany; priesnitz@bnitm.de; 5 Department of Tropical Medicine and Infectious Diseases, Center of Internal Medicine, University Medicine Rostock, Ernst-Heydemann-Strasse 6, 18055 Rostock, Germany; emmerich@bnitm.de; 6 Faculty of Medicine, University “Hasan Prishtina”, 10000 Pristina, Kosovo; salih_ahmeti@hotmail.com; 7 Department of Virology, Bernhard Nocht Institute of Tropical Medicine, 20359 Hamburg, Germany

**Keywords:** Crimean–Congo hemorrhagic fever, CCHF, Kosovo, pregnancy, HELLP syndrome

## Abstract

Crimean–Congo hemorrhagic fever (CCHF) is fatal in 10 to 40% of cases. It is caused by CCHF virus (CCHFV). Symptoms include fever, headache, myalgia, and often hemorrhage and other complications. This report shows that CCHF may resemble HELLP syndrome (hemolysis, elevated liver enzymes, low platelets). We report CCHF in a pregnant mother with fever and suspected HELLP syndrome, who survived, and her infant (week 36), who died six days after C-section. The high CCHF viral load and bacterial sepsis may have jointly contributed to the death of the infant. CCHF should be considered as a differential diagnosis of HELLP syndrome in regions where this viral disease is endemic.

## 1. Introduction

Crimean–Congo hemorrhagic fever (CCHF) is an acute tick-borne febrile zoonosis caused by CCHF virus (CCHFV), an RNA virus of the genus Orthonairovirus, order Bunyavirales, family Nairoviridae [1]. Initial symptoms include fever, headache, and myalgia. Within a few days, petechiae, epistaxis, and gingival bleeding may appear. CCHF is endemic in Kosovo, and a strain named Kosovo Hoti, which is suspected to be more pathogenic than strains from other regions, has been isolated in the country [2,3]. We report a case of CCHF in a pregnant mother with suspected HELLP syndrome (hemolysis, elevated liver enzymes, low platelet count) and in her newborn child in Kosovo.

## 2. Patients and Methods

The clinical course and laboratory data of a surviving pregnant mother with CCHF and her newborn infant who died from CCHF six days after delivery by cesarian section are presented and analyzed in a descriptive manner. The CCHF viral loads were measured by a commercial Polymerase Chain Reaction (PCR) testing kit (Realstar CCHFV RT-PCR, Altona Diagnostics GmbH, Hamburg, Germany) in the National Institute of Public Health of Kosovo. The mother has given written informed consent for a case report in a scientific journal.

### 2.1. Case Presentation 1: Pregnant Mother with Suspected HELLP Syndrome and CCHF

Four days after a tick bite, a 29-year-old farm worker, a primigravida in her 36th week of pregnancy, developed fever, chills, loss of appetite, myalgia, backaches, and dizziness (day 0 of disease: 12 June 2013). On day 4, she was admitted to the ObGyn ward of a local hospital because her symptoms had not improved. On day 7, she self-discharged against medical advice, but was brought back by family members on day 8 because she had lost consciousness.

Upon readmission, a generalized epileptic seizure was observed. Clinical examination revealed minimal peripheral edema, but no other pathological findings. Her blood pressure was 130/90 mm Hg. Magnesium and diazepam were given as secondary prophylaxis against seizures. Since HELLP syndrome was suspected, a cesarian section was performed under general anesthesia, following standard antiseptic precautions. Placental abruption was not seen. Mother and child were transferred to the University Clinical Center of Kosovo in Prishtina on the same day.

Upon arrival at the UCC, the mother was unconscious and tachypneic. Her blood pressure was 93/54 mmHg, heart rate was 122/min, and oxygen saturation was 97% (room air). Auscultation of the lungs revealed fine crepitations. Laboratory parameters showed massive elevations of transaminase and LDH levels, leukocytosis, and anemia, all of which are also seen in HELLP syndrome, and elevated renal function parameters (see Table 1).

Since a cerebral CT scan suggested mild cerebral edema, intravenous mannitol and dexamethasone were initiated. Abdominal sepsis was suspected, and ceftriaxone, gentamycin, and metronidazole were started. In addition, the patient received fluids, blood transfusions, fresh frozen plasma, ergometrin, and ephedrine. In spite of these measures, her clinical state deteriorated, and she had to be intubated and transferred to the ICU the same day, where she required vasopressor support at least intermittently. One day later, day 10 after disease onset, abdominal ultrasound revealed fluid in the abdominal cavity. Cerebrospinal fluid analysis gave normal results for cell count and protein content. By day 13, the amount of intraabdominal fluid increased, and the patient was re-operated due to massive intraperitoneal bleeding and intramuscular hematomas in the abdominal wall.

Testing for CCHF, brucellosis, and Hantaan was initiated on day 10 after disease onset. The rt-PCR result for CCHF virus RNA came back positive on day 13, with a viral load of 3.18 × 10^4^ copies per mL of serum, confirming the diagnosis of CCHF. Sequencing identified the CCHFV strain as Kosovo Hoti. No CCHF viral RNA was detected in the urine, abdominal fluid, or breast milk. Tests for Hantaan virus RNA, antibodies against Brucella, and HBs-antigens gave negative results. As a consequence of these results, therapy with i.v. ribavirin was started the same day (17 mg/kg i.v. loading dose, then four days of 17 mg/kg 4 times daily, then six days of 8 mg/kg 3 times daily, according to the WHO recommendations in effect at the time).

On day 18, the patient was extubated. However, one day later, she developed bleeding signs (epistaxis, gingival bleeding, hemoperitoneum, metrorrhagia, hematuria, and subcutaneous hematomas) and had to be re-intubated for a day because of respiratory failure. During the following days, her situation stabilized. Eventually, she made a full recovery, and was discharged from the hospital on day 39.

### 2.2. Case Presentation 2: Newborn Infant with CCHF, Meconium Aspiration Syndrome, and Probable Bacterial Sepsis

On June 20, 2013, a female fetus in her 36th week was delivered by cesarean section, after the mother described above had suffered epileptic seizures and possibly HELLP syndrome. The mother had been ill for 9 days before delivery, suffering from what turned out to be CCHF. The infant measured 49 cm and weighed 2659 g. The AGPAR score was 0–3. The amniotic fluid was meconium-stained, and the infant, who had aspirated meconium, was afebrile, but asphyxic, cyanotic, bradycardic, and hypotensive, displaying pronounced intercostal and subcostal retractions. In addition, signs of neurological impairment (fasciculations, hyporeflexia) were seen. Direct bleeding was not observed, but the abdomen was distended. Mechanical ventilation was started immediately, and together with her mother, she was transferred to the University Clinical Center of Kosovo in Prishtina, where antibiotic treatment for suspected neonatal sepsis was initiated.

A chest X-ray revealed pneumonia. The laboratory results showed leukocytosis with left shift, thrombocytopenia, and elevated levels of liver transaminases and creatine kinase (Table 2). A blood culture taken upon arrival at the UCC yielded Acinetobacter baumanni 48 h later, resistant to all betalactams tested, gyrase inhibitors, chloramphenicol, and trimethoprim, but sensitive to tetracyclin. Echocardiography showed insufficiency of the tricuspidal and the mitral valve, together with an open foramen ovale. The brain parenchyma was found to be slightly hypoechogenic on CNS ultrasound. Pulmonary hypertension developed on day 4 after delivery. PCR testing of serum obtained on day 5 (i.e., 13 days after the mother developed symptoms) revealed a CCHF viremia of 1.52 × 10^9^ copies per mL. Unfortunately, the infant died on day 6, the same day the PCR result for CCHF came back positive.

## 3. Discussion

Outcomes of CCHF in pregnant mothers and their infants range from full recovery to death. While case fatality rates of CCHF may vary between 10 and 40% in the general population, they can be as high as 34% in pregnant women, and 59% in fetal cases [4]. CCHF can be transmitted from mother to child both vertically and nosocomially [5], and the case fatality rate of nosocomially acquired CCHF probably exceeds 30% [6].

This is the first report of maternal and fetal CCHF in Kosovo. The infant had been delivered by cesarian section because the combination of hemolytic anemia, elevated liver transaminases, and low platelet counts led the clinicians to suspect HELLP syndrome [5]. However, these three findings are also common in CCHF [2], and CCHF was diagnosed only after the cesarean section, both in the mother and in the newborn baby. CCHF infection might trigger HELLP syndrome in pregnant women, and both HELLP syndrome and CCHF are characterized by microvascular damage and organ failure [5]. A likely pathomechanism would be apoptosis of the endothelial and parenchymal cells [7].

The fact that the mother survived but her newborn infant died may be a consequence of their respective viral loads: 3.14 × 10^4^ copies per mL in the mother and 1.52 × 10^9^ copies per mL in the newborn baby. A study of CCHF in Kosovo found that viral loads above 10^8.5^ copies per mL are strongly associated with a fatal outcome [2], probably reflecting the failure of the immune system to control viral replication. The samples for the viral loads had been taken on the same day from the mother and the infant, 13 days after the onset of symptoms in the mother. Since the infant was already symptomatic at delivery, she may have contracted the disease vertically, several days before being delivered by cesarean section.

CCHF infection probably causes death via extensive cellular damage. Glial cells are among those cells susceptible to infection with CCHF virus [8], and the hypoechogenic brain parenchyma of the infant raises the possibility of central nervous system involvement. In CCHF, CNS involvement is a risk factor for death, independent of bleeding complications [2].

On the other hand, the combination of meconium aspiration, pneumonia, and Acinetobacter baumanni bacteriemia suggests that bacterial sepsis contributed to the death of the newborn infant. Acinetobacter baumannii is typically resistant to multiple antibiotics, which often leaves clinicians without effective treatment options. However, this report ultimately cannot ascertain the contribution of Acinetobacter to the deadly outcome in the case of the infant.

A study from Turkey has described five cases of CCHF in pregnant women at the gestational ages of 8, 18, 20, 21, and 32 weeks [9]. Only in the youngest gestational age (8 weeks) was spontaneous abortion observed, while in the other four cases, the mothers gave birth to healthy children. A later analysis of CCHF cases from Russia, Kasakhstan, Turkey, former Yugoslavia (not Kosovo), Iran, Iraq, Bulgaria, and Mauretania reported 1 maternal death and 6 fetal deaths in 11 pregnancies with gestational ages between 0 and 20 weeks, in contrast to 8 maternal deaths and 9 fetal/neonatal deaths in 18 pregnancies with gestational ages between 20 and 40 weeks [4]. These data appear to argue against a major role of gestational age in the risk of fetal death in maternal CCHF infection. The high pathogenicity of the Kosovo Hoti strain of CCHF that is prevalent in Kosovo may also have contributed to the adverse fetal outcome [2,3].

It remains uncertain whether the infant had been infected with CCHF virus vertically or nosocomially (i.e., during the cesarean section), since both transmission modes are possible in CCHF. No virus was detectable in the abdominal fluid of the mother. Unfortunately, amniotic fluid could not be tested. The serum sample of the infant, which showed a high viral load, had been obtained five days after the cesarean section, but probably more than five days after disease onset in utero. Thus, in this case of maternal and neonatal CCHF, neonatal death may have been caused both by the viral infection itself, as well as by complicating bacterial sepsis.

## 4. Conclusions

CCHF may present with findings that are characteristic of HELLP syndrome. Therefore, if HELLP syndrome is suspected in a region known to be endemic for CCHF, rapid testing for CCHF should be initiated. Precautions, including barrier nursing, should be implemented to protect healthcare workers until CCHF has been excluded. Also, this case illustrates that life-saving obstetric care can be safely provided in cases of CCHF.

## Figures and Tables

**Table 1:** Laboratory parameters of the mother after transfer to the University Clinical Center (UCC) of Kosovo in Prishtina. Unfortunately, laboratory results from the referring hospital that had performed the cesarian section were not available.

Days After Disease Onset	Day 9 (Arrival at UCC)	Day 9 (Transfer to ICU)	Day 10	Day 13	Day 18	Day 19	Day 25		
Parameter								Unit	Normal Values
									</ = 1:20
Anti-CCHF IgG IFT	1:160						1:10,240 (Day 36)		</ = 1:20
Anti-CCHF IgM IFT	1:80						1:10,240 (Day 36)		3.5–6.0
Erythrocyte Count	3.52	3.05	3.1	2.3	4.1	3.5	3.1	×10^12^/L	95–150
Hemoglobin	95	82	84	68	121	94	95	g/L	3.5–10.0
Leukocyte Count	26.6	24.2	22.4	13.8	14.6	14.1	9.5	×10^9^/L	150–450
Platelet Count	101	149	156	73	52	81	207	×10^9^/L	</ = 1:20
ASAT (GPT)	2050		1097	65	108	62	33	IU/L	<50
ALAT (GOT)	470		253	229	86	55	24	IU/L	<50
LDH	2733		1511					IU/L	<240
Total Bilirubin	60.5		61.1					µmol/L	<20
Prothrombin Time			37.5	39.1			Normal	s	<11
PTT			85				Normal	s	20–40
Activated PTT			52				Normal	s	20–40
Albumin	23.3		21.4	23				g/L	35–45
Creatinine	126		129	114	59	58		µmol/L	<90
Urea	9.8		10.4	10.1	6.1	4.8		mmol/L	<7
Glucose	5.5			9.9	3.1	6.6		mmol/L	3.5–7.5
CRP	14.3		16.4					mg/L	<5
Procalcitonin			0.68					ng/mL	<0.5
Na	143							mmol/L	135–145
K	4.2							mmol/L	3.5–5.2

**Table 2:** Laboratory parameters of the baby girl after transfer to the University Clinical Center (UCC) of Kosovo in Prishtina.

	Days Since Cesarian Section				
Parameter	Day 0	Day 3	Day 6	Unit	Normal Range
Erythrocyte Count	4.12	4.7	3.9	×10^12^/L	3.5–6.0
Hemoglobin	142	143	136	g/L	95–150
Hematocrit			39.5	%	30–40
Leukocyte Count	30; Left Shift	15.3	17.1	×10^9^/L	3.5–10.0
Platelet Count	108	46	49	×10^9^/L	150–450
AST (GPT)			156	IU/L	<50
ALT (GOT)			56	IU/L	<50
Creatine Kinase			313	IU/L	<190
Total Bilirubin			79.3	µmol/L	<20
Direct Bilirubin			53.0	µmol/L	<6
Prothrombin Time			44.8	s	<11
Albumin			26.1	g/L	35–45
Creatinine			85.5	µmol/L	<90
Urea			9.1	mmol/L	<7
Glucose	3.9			mmol/L	3.5–7.5

## Data Availability

All clinical data of the case are included in this publication.

## Abbreviations

CCHF	Crimean–Congo Hemorrhagic Fever
HELLP Syndrome	Hemolysis, Elevated Liver Transaminases, Low Platelet Count
IFT	Immunofluorescence Test
ASAT	Aspartate Amino Transferase
ALAT	Alanine Amino Transferase
LDH	Lactate Dehydrogenase
PTT	Partial Thromboplastine Time
